# Supplementary material for: The long non-coding RNA Dali is an epigenetic regulator of neural differentiation
Source: eLife. 2014 Nov 21;3:e04530. doi: 10.7554/eLife.04530 (PMC4383022; doi:10.7554/eLife.04530)
Supplement: Supplementary file 1. — Oligonuleotides. DOI: http://dx.doi.org/10.7554/eLife.04530.016 [file elife04530s001.docx]

| Dali qPCR |  |
| --- | --- |
| linc8 for1821 | CCTCCGATCCTGCAGTTAGC |
| linc8 rev1912 | ACAGCAGTTCAGCAGCAAGAAG |
|  |  |
| AK011913 qPCR |  |
| linc8bF1 | AGAAGGCCTCCCTGAAAGTG |
| linc8bR1 | ACCAAGATCCCTGTCACACAC |
|  |  |
| Linc-Brn1a qPCR |  |
| linc8uF1 | CGGGACTGTAAGGCGGATA |
| linc8uR1 | AATTGGCAGGAGGAGCATC |
|  |  |
| Pou3f3 qPCR |  |
| Pou3f3 for1025 | TGGCTCTGGGTACGCTCTATG |
| Pou3f3 rev1124 | GGCTTGAGCTTGCACATGTTC |
|  |  |
| Dali KD oligos |  |
| Linc8sh74For | GCTTGGATTCAGTTCCTATTCAAGAGACAGGAACTGAATCCAAGCCTTTTTC |
| Linc8sh74Rev | TCGAGAAAAAGGCTTGGATTCAGTTCCTGTCTCTTGAATAGGAACTGAATCCAAGC |
| Linc8sh862For | AGCTACTGATGAGACATATTCAAGAGACATGTCTCATCAGTAGCTCTTTTTC |
| Linc8sh862Rev | TCGAGAAAAAGAGCTACTGATGAGACATGTCTCTTGAATATGTCTCATCAGTAGCT |
| Linc8sh906For | ATCAACACTCATCAAGAATTCAAGAGACTCTTGATGAGTGTTGATCTTTTTC |
| Linc8sh906Rev | TCGAGAAAAAGATCAACACTCATCAAGAGTCTCTTGAATTCTTGATGAGTGTTGAT |
| Linc8sh1000For | TGGTCTACAGTAGAGAATTTCAAGAGACTTCTCTACTGTAGACCACTTTTTC |
| Linc8sh1000Rev | TCGAGAAAAAGTGGTCTACAGTAGAGAAGTCTCTTGAAATTCTCTACTGTAGACCA |
|  |  |
| Pou3f3 KD oligos |  |
| Pou3f3sh1941f | AAGGGTGAAGATGCCTAATTCAAGAGACTAGGCATCTTCACCCTTCTTTTTC |
| Pou3f3sh1941r | TCGAGAAAAAGAAGGGTGAAGATGCCTAGTCTCTTGAATTAGGCATCTTCACCCTT |
| Pou3f3sh2687f | CGGATGTGATTATGTAATTTCAAGAGACTTACATAATCACATCCGCTTTTTC |
| Pou3f3sh2687r | TCGAGAAAAAGCGGATGTGATTATGTAAGTCTCTTGAAATTACATAATCACATCCG |
| Pou3f3sh2765f | CAATATAGAATCGTTAGATTCAAGAGACCTAACGATTCTATATTGCTTTTTC |
| Pou3f3sh2765r | TCGAGAAAAAGCAATATAGAATCGTTAGGTCTCTTGAATCTAACGATTCTATATTG |
| Pou3f3sh3012f | AGTAGTGATTCCGTAAGATTCAAGAGACCTTACGGAATCACTACTCTTTTTC |
| Pou3f3sh3012r | TCGAGAAAAAGAGTAGTGATTCCGTAAGGTCTCTTGAATCTTACGGAATCACTACT |
|  |  |
| Dali expression  plasmid |  |
| Linc8lforEcoRI | AGACGAATTCGAGTAGTATCAGTATAGATAAGAG |
| Linc8lrevXhoI | AGACCTCGAGGTATTCTTTCCTTTCCCCTTTATT |
|  |  |
| FLAG-Pou3f3 expression vector |  |
| PouFLAGrev1 | CTGCGCCCCGGCATTCACTGCTCTAGAAGAC |
| PouFLAGrev2 | CGGCATTCACTGCACGCTGGTCTGATCTAGAAGAC |
|  |  |
| CHART |  |
| RNase H sensitivity |  |
| qPCR |  |
| linc8-367F | CAGAGATCTGCCTACCTCTTCTG |
| linc8-367R | GAGTCACCTCCCCAAAATCTATAA |
| linc8-855F | GCACTCCTTCATGGCCTCT |
| linc8-855R | GTCAAGGCAGGAACTCAACC |
| linc8-1223F | CAAGGACAAATGAGCTGCCTA |
| linc8-1223R | GTCACAGGGAACTCCACCAT |
| linc8-1594F | CCTCTGTCTCTGTGTACCAGGATT |
| linc8-1594R | GGCCATAACTACCTAAGCATCATT |
| linc8 for1821 | CCTCCGATCCTGCAGTTAGC |
| linc8 rev1912 | ACAGCAGTTCAGCAGCAAGAAG |
| linc8-2025F | CTGGAGCCCCAGAGTCTTAG |
| linc8-2025R | TCCTTCACTAGGCAATTTGGA |
| linc8-2365F | CCTGTGGTCTGCACTCACTG |
| linc8-2365R | CTGGTGGGAAGCATCTGAG |
| linc8-357595F | CTCTTCTGCTCCACCTCCAC |
| linc8-357595R | TGTGCATGCCTGGTTTATGT |
| linc8-12501463F | CCCTGTGACTGTGGGATTCT |
| linc8-12501463R | TGTGCAGTGTTCAGGCTAGG |
| linc8-8861089F | CTGCCTTGACTTTCCCTCAG |
| linc8-8861089R | TGGTCAAGGGGCATTTTAAG |
|  |  |
| RNase H mapping |  |
| linc8FISH7 | AAAAACTATAGCTGGGCAGG |
| linc8FISH8 | GTCACCTCCCCAAAATCTAT |
| linc8FISH9 | GGAGAGTTGCAAATGCTCTT |
| linc8FISH10 | TAGGAACTGAATCCAAGCCT |
| linc8FISH11 | ATGATTGTGAGCCACCATGT |
| linc8FISH13 | GTGCATGCCTGGTTTATGTA |
| linc8FISH14 | GGTGTTTGCCTGCACAATAT |
| linc8FISH21 | AGAACACTCTATCACTGAGG |
| linc8FISH22 | CGAGGGTTTACTTCATCTAC |
| linc8FISH23 | TCATGACCAAGGGCAGTTTA |
| linc8FISH24 | GGGGTTCCATTGCTATGATA |
| linc8FISH25 | TGAAGCAAACCTGTCTGGAA |
| linc8FISH26 | ACTAGGAAGGCTTTGCTGAA |
| linc8FISH27 | GACTCTTGCATTCTCTACTG |
| linc8FISH28 | AATTGGTGTCTGTGTGCAGT |
| linc8FISH29 | GGTAGATTGTCACAAGTTCC |
| linc8FISH30 | AATCCTGGTACACAGAGACA |
| linc8FISH31 | TGAAGAAGAATGTGTGCCCA |
| linc8FISH32 | GCCATAACTACCTAAGCATC |
|  |  |
| C-oligos |  |
| linc8-C-oligo10 | TAGGAACTGAATCCAAGCCTCCTTG-HEG-BIOTIN-TEG |
| linc8-C-oligo11 | GGTGGAGGATTTTTCTCAACTGAGG-HEG-BIOTIN-TEG |
| linc8-C-oligo25 | TGAAGCAAACCTGTCTGGAAGCACA-HEG-BIOTIN-TEG |
| linc8-C-oligo27 | GACTCTTGCATTCTCTACTGTAGAC-HEG-BIOTIN-TEG |
| linc8-C-oligo30 | AATCCTGGTACACAGAGACAGAGGC-HEG-BIOTIN-TEG |
| cons1-1 | GCTTTTCATCACCCATCTGGCTCTC-HEG-BIOTIN-TEG |
| cons1-2 | GACTCTGCTTTCAAGAAGTGACAGC-HEG-BIOTIN-TEG |
| cons2-1 | GAAGCATCTGAGAAGTAGCCTGACC-HEG-BIOTIN-TEG |
| cons2-2 | TCAAAATGGAGTTCACTGCGGCTGG-HEG-BIOTIN-TEG |
| noncons1 | TTCCAAAGGGACTTTGGAACAGGTG-HEG-BIOTIN-TEG |
| lacZ | GACGGCCAGTGAATCCGTAATCATG-HEG-BIOTIN-TEG |
| sense-linc8 | GTCTACAGTAGAGAATGCAAGAGTCCATCC-HEG-BIOTIN-TEG |
|  |  |
| CHART-seq peak validation |  |
| Tgfb3chartF2 | CCCATTGGCACTCACTCC |
| Tgfb3chartR2 | GCAAACAGCAGGAGGCTAAA |
| E2F2chartF1 | TTCCCACCAGGGTCTCAG |
| E2F2chartR1 | CTGGAGCGAAAGGGGAAG |
| ActBchartF1 | GCCATCCTATCCCAAGCATA |
| ActBchartR1 | TCTTCTTGCAACACCTCCAG |
| Aimp2chartF1 | TGGGTGTTCCAGGAGACG |
| Aimp2chartR1 | ACCTCACGCCTTCGGTTT |
| DolkchartF1 | TGTAGCAGATGTTTGCCCTCT |
| DolkchartR1 | CAAGATGGGGCTGTCAAGAT |
| Ube2cchartF1 | TTTAATGGTCGGCGTCGT |
| Ube2cchartR1 | AAAGCTGCCATTAACTAACGAATC |
| Dpm2chartF1 | GACCCTCCGTTAGTGCTTGT |
| Dpm2chartR1 | TGGCCTGGAGAAAAATAAAGG |
| Phox2bchartF1 | AGTGGGGAGATGTGCACTG |
| Phox2bchartR1 | GCTCGGCTGGTAGTAAGGAG |
| Pax6chartF1 | CTCATTTCCCGCTCTGGTT |
| Pax6chartR1 | CATTAGCGAAGCCTGACCTC |
| AK146698chartF1 | CGGAAAGAGTGGAAGTCGAT |
| AK146698chartR1 | CCCTCAGTGAACTCCTCCAC |
| Pou3f3 for1025 | TGGCTCTGGGTACGCTCTATG |
| Pou3f3 rev1124 | GGCTTGAGCTTGCACATGTTC |
|  |  |
| Dali 5′ RACE |  |
| Reverse GSP |  |
| Linc8RevGSP | TGGGGGACGGTGTTTGCCTGCACAAT |
| Linc8RevGSP2 | GCAGGAGCTGATGCAGAGGCCATGA |
|  |  |
| Forward GSP |  |
| Linc8ForGSP | CACTCACTGCCCTGGCTGCAGGTTGAA |
| Linc8ForGSP2 | GGTCCAACTCGTTATGGCCCCAGGTGTT |
|  |  |
| Nested |  |
| Linc8NestedRev | TGTGCATCATGTGCATGCCTGGTT |
| Linc8NestedFor | GCTTCCCACCAGCCGCAGTGAACTC |
|  |  |
|  |  |
| 3C |  |
| PouR1 | CAAGGAGCTGTACAACCACAG |
| PouR3 | AAGATGGAACACAGATTTCATTG |
| PouR4 | CGGGGTGAAAAGATACCATC |
| PouR5 | TTTTGATTTGTGCTCGTCTGA |
| PouR6 | TCAAAAACGTACTCTGCTACCC |
| PouR7 | AAGACTTCGGAGCTAGTTTTGG |
| PouR9 | GGCTTCTCTGCAGCCTAGAT |
| PouR13 | TCTTCAGACACCCCAGAAGAG |
| PouR16 | GCAGACAAAGTGACAAGTCAGA |
| PouR19 | TCCCTCTTGTTAGAACCCAGAT |
| PouR20 | CACTCAACGTGGTTTATGTGTT |
| PouR21 | GGGCATTTTAGTAATCGGATAAT |
| PouR22 | TGGTTTCTCCTTATCTTCAGGAC |
| PouR23 | AGGTGACTTGGTGGTCCAGT |
| PouR24 | CAGAGAGAGTTCCAAAACAACC |
| GAPDH 1 | GTTTCCATAGGACCTGCTGCG |
| GAPDH 2 | GTTTTACACTGGGCACTTGAGGTC |
| hlinc82R1 | CCCCAGAGAAAGTTGAGCAGCCCAAAG |
| hlinc82Ln | GGGCTGCTCAACTTTCTCTGGGGTCA |
| hlinc82L1 | TGGGGTTCAAGCTCTTCAGCCCATGT |
| hlinc83Rn | CTCCAGACCGCTGGGACCATGGATA |
| hlinc83R1 | CAGCGGCAAGCACCAGGTGAGACTA |
| hlinc83Ln | GGCTCCCACAGCCACAGTCAACTCC |
| hlinc83L1 | TGGCCTCTGTGTCTGTGCCTGCAAT |
| hlinc84Rn | CAAGGGGCCCCGTTATCACCAATGT |
| hlinc84R1 | CAATGACAGCCCTGGAAAGTGAACCA |
| hlinc84L1 | CGGGGCCCCTTGACTTGCTATTGAC |
|  |  |
|  |  |
| CTCF ChIP |  |
| THChIPF1 | GTCAGCCAACATGGGTACG |
| THChIPR1 | GTGGCCTCACACAGAGACTG |
| Tgfb3chartF2 | CCCATTGGCACTCACTCC |
| Tgfb3chartR2 | GCAAACAGCAGGAGGCTAAA |
| Creb3l2ChIPF1 | GCGGAGAGGGTCTTTTACG |
| Creb3l2ChIPR1 | AGCACCCTATTGGTCCATTG |
| Slc1a4ChIPF1 | CTAGGGAGTCCGGAACGAC |
| Slc1a4ChIPR1 | GCCTACAGAGCCCTTAGAAGAA |
| GanabChIPF1 | CCTGGGCCAGTTTCATTAGT |
| GanabChIPR1 | TGCAGACTGTGAACTTACTGGAC |
| Zfp954ChIPF1 | GGGGACATGAACGTCTGC |
| Zfp954ChIPR1 | ATCGCCCACCTACCTGAGTT |
| AcheChIPloF1 | CACATTTCAGGGAGTTGGTTC |
| AcheChIPloR1 | TTGTCGCCGAACATACTTAGG |
| Linc8 for1821 (DaliChIPF) | CCTCCGATCCTGCAGTTAGC |
| Linc8 rev1912 (DaliChIPR) | ACAGCAGTTCAGCAGCAAGAAG |
| Pou3f3 for1025 (Pou3f3ChIPF) | TGGCTCTGGGTACGCTCTATG |
| Pou3f3 rev1124 (Pou3f3ChIPR) | GGCTTGAGCTTGCACATGTTC |
| linc5F1 | GGCACCTTCCTCCTAAGACAC |
| linc5R1 | CCATTCCCTATGTTGTCACCTC |
| GAPDH 1 | GTTTCCATAGGACCTGCTGCG |
| GAPDH 2 | GTTTTACACTGGGCACTTGAGGTC |
|  |  |
| POU3F3 ChIP |  |
| Chga3ChIPF1 | TGAATGTGAAATTTGTTCTGATTCTT |
| Chga3ChIPR1 | CCCCTCGGGAGTGAAAAA |
| Fam92bChIPF1 | GGAGATGGGGCTCATTCA |
| Fam92bChIPR1 | GGTACAGGAGGCGGATTCTC |
| Prr11ChIPF1 | CGCAGTGGAAGCTTTTATGTC |
| Prr11ChIPR1 | AGTTTGCGATTGGTGTACTCG |
| Gins1ChIPF1 | CCAGTGCACTTCTATTGGTTGA |
| Gins1ChIPR1 | TCAGAGTCGCACAACACCA |
| Itga2ChIPF1 | CAAACTCCGGATCTGTCCTG |
| Itga2ChIPR1 | TGGATCTTGACTCCTTTTTCAGA |
| Lgi1ChIPF1 | CCAAAATCCACGGATCTCAC |
| Lgi1ChIPR1 | AACTTGGGCAGATGGTTCC |
| Galnt5ChIPF1 | GGCACAAATACTTTGCTGTGAA |
| Galnt5ChIPR1 | TCAGCTCTTACTGACTGCCTGT |
| DbhChIPF1 | CCCCACTGGACAGGCATA |
| DbhChIPR1 | GGCTGAGATGAGCTTGCAT |
| Foxd1ChIPF1 | TGGGCTTCAGTCTCCAACTC |
| Foxd1ChIPR1 | AATCGGCGTGTAGGAAGAGA |
| Arhgap18ChIPF1 | ATGCCTTCCTTGAGTGATGC |
| Arhgap18ChIPR1 | TTTCCCTTCCCCATGTGTTA |
| Angpt2ChIPF1 | ATGTCAGAGGCACCACCCTA |
| Angpt2ChIPR1 | GCTTAGCCTACAAACGAGCAG |
|  |  |
|  |  |
| Dali KD array validation oligos |  |
| Rrm2F | GCAGCCAGTGATGGAATTG |
| Rrm2R | GGAACCTGCACCTCCTGAC |
| Anxa2F | GGAAATATGGCAAGTCCCTGT |
| Anxa2R | TCTGGTAGTCACCCTTGGTGT |
| NaspF | TGGGTGACATTCCAGCAG |
| NaspR | GCCGTTTCTCCATACTTCTTACC |
| HellsF | TGATCATTTATGACAGTGATTGGAA |
| HellsR | TTGTCTGACCAATTCTATGACATCTA |
| Mcm3F | CAGGACTCCCAGAAAGTGGA |
| Mcm3R | TAAGAGGGCCGCCTTAAAA |
| GanabF | TGATCCATGAAGTCACCAAGG |
| GanabR | CCTGATCCGAGTCATGTTCTT |
| AcheF | GCTGTCACTGTCGGCTCA |
| AcheR | AGGACAGGCTGGTGTCTGA |
|  |  |
|  |  |
| COBRA |  |
| Fbn1COBRAF3 | GGTAGAGAGGGTTAGTTGTAGATGTT |
| Fbn1COBRAR3 | AATTAAAACAAAAACTATAAATACCACAAA |
| Nos1COBRAF1 | AAAAGATGTATGTTTTTAAGTTTAGAG |
| Nos1COBRAR1 | AAAAACTACCAACTTCCCCTTAC |
| Dlgap5COBRAF2 | TTAGGGATTTAAGTGTGAGGTGTAAG |
| Dlgap5COBRAR2 | AAAAACACCTTAAATAAATAAAAACC |
| Hmgb2COBRAF1N | GGTAAGGTGAGGGATTTTGGT |
| Hmgb2COBRAR1 | CCCCTAATAATTTAAAAATAAATAAAATAA |
|  |  |
|  |  |
| Nos1 alt TSS |  |
| Nos1Ex1F | GATCCACAGCCCTGGAACT |
| Nos1Ex1R | AGATGCAGAGGTGATGTAGGG |
| Nos1Ex2F | TCGCTTCCTTAAGGTCAAGAAC |
| Nos1Ex2R | CTCTGTGCACCCCGTTTC |
| Nos1F3 | CATCAGGCACCCCAAGTT |
| Nos1R3 | CAGCAGCATGTTGGACACA |


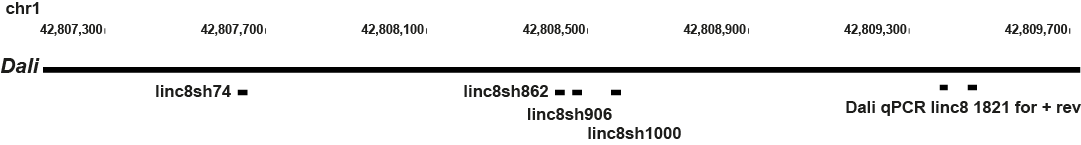


Schematic representation of qPCR primers used to quantify Dali expression and shRNA oligos used for its knockdown.
