# Supplementary material for: The long non-coding RNA Dali is an epigenetic regulator of neural differentiation
Source: eLife. 2014 Nov 21;3:e04530. doi: 10.7554/eLife.04530 (PMC4383022; doi:10.7554/eLife.04530)
Supplement: Supplementary file 6. — Motif discovery. DOI: http://dx.doi.org/10.7554/eLife.04530.021 [file elife04530s006.docx]

| **Motif group** | **No. motifs** | **Algorithm** | **Representative motif** | **E value** | **No. peaks** | **Transfac motif matches** | **DNMT1 protein-protein interaction** |
| --- | --- | --- | --- | --- | --- | --- | --- |
| 1 |  | MEME | 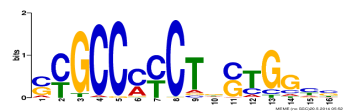 | 1.9e-175 | 332 | [MAZ (M02023)](https://www.cgat.org/downloads/myRInhR8rR/dali/motif_analysis/dali_lacz_input.all.500/meme_tomtom_out/tomtom.html#match_q_1_t_1_M02023), [Zfp281 (M01597)](https://www.cgat.org/downloads/myRInhR8rR/dali/motif_analysis/dali_lacz_input.all.500/meme_tomtom_out/tomtom.html#match_q_1_t_1_M01597), [KROX (M00982)](https://www.cgat.org/downloads/myRInhR8rR/dali/motif_analysis/dali_lacz_input.all.500/meme_tomtom_out/tomtom.html#match_q_1_t_1_M00982) | n/a |
| 2 |  | MEME | 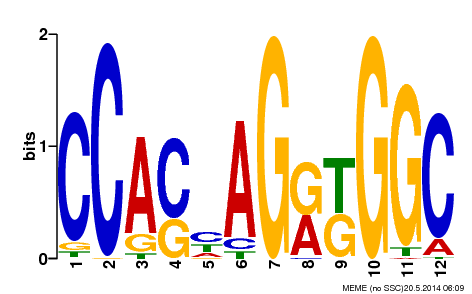 | 3.1e-062 | 125 | [CTCF (M01259, M01200)](https://www.cgat.org/downloads/myRInhR8rR/dali/motif_analysis/dali_lacz_input.all.500/meme_tomtom_out/tomtom.html#match_q_2_t_1_M01259), [MATH1 (M01716)](https://www.cgat.org/downloads/myRInhR8rR/dali/motif_analysis/dali_lacz_input.all.500/meme_tomtom_out/tomtom.html#match_q_2_t_1_M01716) | CTCF (Hervouet et al, 2012) |
| 3 |  | MEME | 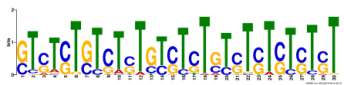 | 4.8e-122 | 32 | [SMAD3 (M00701)](https://www.cgat.org/downloads/myRInhR8rR/dali/motif_analysis/dali_lacz_input.all.500/meme_tomtom_out/tomtom.html#match_q_4_t_1_M00701), [GAGA (M00723)](https://www.cgat.org/downloads/myRInhR8rR/dali/motif_analysis/dali_lacz_input.all.500/meme_tomtom_out/tomtom.html#match_q_4_t_1_M00723) | n/a |
| 4 |  | MEME | 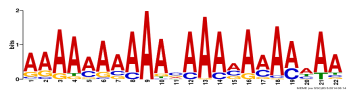 | 2.3e-045 | 41 | [FOXP1 (M00987)](https://www.cgat.org/downloads/myRInhR8rR/dali/motif_analysis/dali_lacz_input.all.500/meme_tomtom_out/tomtom.html#match_q_5_t_1_M00987), [FOXO1 (M01216)](https://www.cgat.org/downloads/myRInhR8rR/dali/motif_analysis/dali_lacz_input.all.500/meme_tomtom_out/tomtom.html#match_q_5_t_1_M01216), [BR-C (M00092)](https://www.cgat.org/downloads/myRInhR8rR/dali/motif_analysis/dali_lacz_input.all.500/meme_tomtom_out/tomtom.html#match_q_5_t_1_M00092) | n/a |
| 5 |  | MEME | 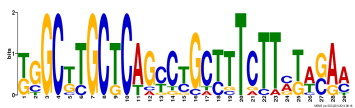 | 5.3e-009 | 10 | [Dde (M00304)](https://www.cgat.org/downloads/myRInhR8rR/dali/motif_analysis/dali_lacz_input.all.500/meme_tomtom_out/tomtom.html#match_q_7_t_1_M00304), [BPC1 (M01126)](https://www.cgat.org/downloads/myRInhR8rR/dali/motif_analysis/dali_lacz_input.all.500/meme_tomtom_out/tomtom.html#match_q_7_t_1_M01126) | n/a |
| 6 |  | MEME | 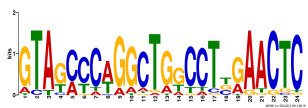 | 5.4e-038 | 23 | [Pax-6 (M00979)](https://www.cgat.org/downloads/myRInhR8rR/dali/motif_analysis/dali_lacz_input.all.500/meme_tomtom_out/tomtom.html#match_q_8_t_1_M00979), [LRH1 (M01142)](https://www.cgat.org/downloads/myRInhR8rR/dali/motif_analysis/dali_lacz_input.all.500/meme_tomtom_out/tomtom.html#match_q_8_t_1_M01142), [ZBED6 (M01598)](https://www.cgat.org/downloads/myRInhR8rR/dali/motif_analysis/dali_lacz_input.all.500/meme_tomtom_out/tomtom.html#match_q_8_t_1_M01598) | Pax-6, LRH1 (Hervouet et al, 2012) |
| 7 |  | MEME | 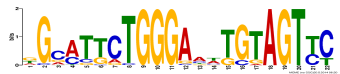 | 5.7e-046 | 24 | [STATx (M00223)](https://www.cgat.org/downloads/myRInhR8rR/dali/motif_analysis/dali_lacz_input.all.500/meme_tomtom_out/tomtom.html#match_q_13_t_1_M00223), [Staf (M00264)](https://www.cgat.org/downloads/myRInhR8rR/dali/motif_analysis/dali_lacz_input.all.500/meme_tomtom_out/tomtom.html#match_q_13_t_1_M00264), [STAT1 (M01823)](https://www.cgat.org/downloads/myRInhR8rR/dali/motif_analysis/dali_lacz_input.all.500/meme_tomtom_out/tomtom.html#match_q_13_t_1_M01823) | STAT1 (Hervouet et al, 2012) |
| 8 |  | DREME | 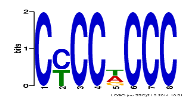 | 7.8e-036 |  | [Zfp281 (M01597)](https://www.cgat.org/downloads/myRInhR8rR/dali/motif_analysis/dali_lacz_input.all.500/dreme_tomtom_out/tomtom.html#match_q_CYCCDCCC_t_1_M01597), [UF1H3BETA (M01068)](https://www.cgat.org/downloads/myRInhR8rR/dali/motif_analysis/dali_lacz_input.all.500/dreme_tomtom_out/tomtom.html#match_q_CYCCDCCC_t_1_M01068), [MAZ (M02023)](https://www.cgat.org/downloads/myRInhR8rR/dali/motif_analysis/dali_lacz_input.all.500/dreme_tomtom_out/tomtom.html#match_q_CYCCDCCC_t_1_M02023) | n/a |
| 9 |  | DREME | 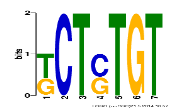 | 8.8e-028 |  | [GAGA (M00723)](https://www.cgat.org/downloads/myRInhR8rR/dali/motif_analysis/dali_lacz_input.all.500/dreme_tomtom_out/tomtom.html#match_q_ACASAGM_t_1_M00723) | n/a |
| 10 |  | DREME | 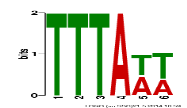 | 7.6e-023 |  | [ML1 (M01193)](https://www.cgat.org/downloads/myRInhR8rR/dali/motif_analysis/dali_lacz_input.all.500/dreme_tomtom_out/tomtom.html#match_q_TTTAWW_t_1_M01193), [Lentiviral (M00318)](https://www.cgat.org/downloads/myRInhR8rR/dali/motif_analysis/dali_lacz_input.all.500/dreme_tomtom_out/tomtom.html#match_q_TTTAWW_t_1_M00318), [PBF (M00355)](https://www.cgat.org/downloads/myRInhR8rR/dali/motif_analysis/dali_lacz_input.all.500/dreme_tomtom_out/tomtom.html#match_q_TTTAWW_t_1_M00355) | n/a |
| 11 |  | DREME | 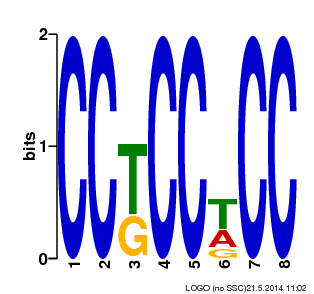 | 3.1e-021 |  | [FPM315 (M01587)](https://www.cgat.org/downloads/myRInhR8rR/dali/motif_analysis/dali_lacz_input.all.500/dreme_tomtom_out/tomtom.html#match_q_CCKCCDCC_t_1_M01587), [ETF (M00695)](https://www.cgat.org/downloads/myRInhR8rR/dali/motif_analysis/dali_lacz_input.all.500/dreme_tomtom_out/tomtom.html#match_q_CCKCCDCC_t_1_M00695) | n/a |
| 12 |  | DREME | 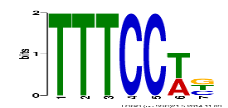 | 1.7e-019 |  | [NFAT3 (M01734)](https://www.cgat.org/downloads/myRInhR8rR/dali/motif_analysis/dali_lacz_input.all.500/dreme_tomtom_out/tomtom.html#match_q_TTTCCWB_t_1_M01734), [NF-AT (M00935)](https://www.cgat.org/downloads/myRInhR8rR/dali/motif_analysis/dali_lacz_input.all.500/dreme_tomtom_out/tomtom.html#match_q_TTTCCWB_t_1_M00935), [PARP (M02027)](https://www.cgat.org/downloads/myRInhR8rR/dali/motif_analysis/dali_lacz_input.all.500/dreme_tomtom_out/tomtom.html#match_q_TTTCCWB_t_1_M02027) | PARP (Hervouet et al, 2012) |
| 13 |  | DREME | 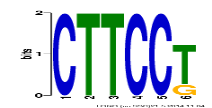 | 1.3e-016 |  | [C-ets-1 (M01870)](https://www.cgat.org/downloads/myRInhR8rR/dali/motif_analysis/dali_lacz_input.all.500/dreme_tomtom_out/tomtom.html#match_q_CTTCCK_t_1_M01870), [Tel-2 (M00678)](https://www.cgat.org/downloads/myRInhR8rR/dali/motif_analysis/dali_lacz_input.all.500/dreme_tomtom_out/tomtom.html#match_q_CTTCCK_t_1_M00678), [ELF1 (M01266)](https://www.cgat.org/downloads/myRInhR8rR/dali/motif_analysis/dali_lacz_input.all.500/dreme_tomtom_out/tomtom.html#match_q_CTTCCK_t_1_M01266) | C-ets-1 (Hervouet et al, 2012) |
| 14 |  | DREME | 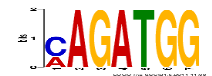 | 2.9e-013 |  | [Tal-1beta:ITF-2 (M00070)](https://www.cgat.org/downloads/myRInhR8rR/dali/motif_analysis/dali_lacz_input.all.500/dreme_tomtom_out/tomtom.html#match_q_CCATCTK_t_1_M00070), [Tal-1beta:E47 (M00065)](https://www.cgat.org/downloads/myRInhR8rR/dali/motif_analysis/dali_lacz_input.all.500/dreme_tomtom_out/tomtom.html#match_q_CCATCTK_t_1_M00065) [Tal-1alpha:E47 (M00066)](https://www.cgat.org/downloads/myRInhR8rR/dali/motif_analysis/dali_lacz_input.all.500/dreme_tomtom_out/tomtom.html#match_q_CCATCTK_t_1_M00066) | n/a |
| 15 |  | DREME | 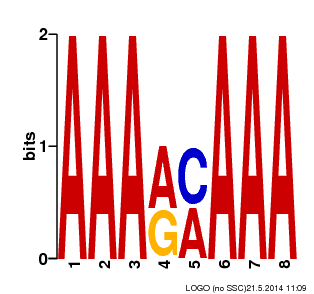 | 7.3e-013 |  | [GT-1 (M01827)](https://www.cgat.org/downloads/myRInhR8rR/dali/motif_analysis/dali_lacz_input.all.500/dreme_tomtom_out/tomtom.html#match_q_AAARMAAA_t_1_M01827), [FOXO1 (M01216)](https://www.cgat.org/downloads/myRInhR8rR/dali/motif_analysis/dali_lacz_input.all.500/dreme_tomtom_out/tomtom.html#match_q_AAARMAAA_t_1_M01216), [HNF3alpha (M00724)](https://www.cgat.org/downloads/myRInhR8rR/dali/motif_analysis/dali_lacz_input.all.500/dreme_tomtom_out/tomtom.html#match_q_AAARMAAA_t_1_M00724) | n/a |
| 16 |  | DREME | 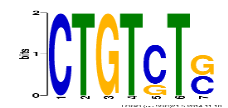 | 4.5e-012 |  | [Smad3 (M01888)](https://www.cgat.org/downloads/myRInhR8rR/dali/motif_analysis/dali_lacz_input.all.500/dreme_tomtom_out/tomtom.html#match_q_CTGTSTS_t_1_M01888), [SMAD (M00974)](https://www.cgat.org/downloads/myRInhR8rR/dali/motif_analysis/dali_lacz_input.all.500/dreme_tomtom_out/tomtom.html#match_q_CTGTSTS_t_1_M00974) | n/a |
| 17 |  | DREME | 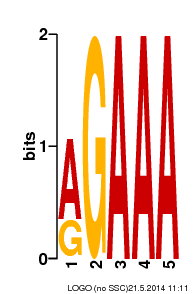 | 2.0e-010 |  | [PARP (M02027)](https://www.cgat.org/downloads/myRInhR8rR/dali/motif_analysis/dali_lacz_input.all.500/dreme_tomtom_out/tomtom.html#match_q_RGAAA_t_1_M02027), [STAT5A (M01890)](https://www.cgat.org/downloads/myRInhR8rR/dali/motif_analysis/dali_lacz_input.all.500/dreme_tomtom_out/tomtom.html#match_q_RGAAA_t_1_M01890), [BPC1 (M01126)](https://www.cgat.org/downloads/myRInhR8rR/dali/motif_analysis/dali_lacz_input.all.500/dreme_tomtom_out/tomtom.html#match_q_RGAAA_t_1_M01126) | PARP (Hervouet et al, 2012) |
| 18 |  | DREME | 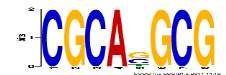 | 2.0e-010 |  | [Zfp206 (M01742)](https://www.cgat.org/downloads/myRInhR8rR/dali/motif_analysis/dali_lacz_input.all.500/dreme_tomtom_out/tomtom.html#match_q_CGCABGCG_t_1_M01742), [NRF-1 (M00652)](https://www.cgat.org/downloads/myRInhR8rR/dali/motif_analysis/dali_lacz_input.all.500/dreme_tomtom_out/tomtom.html#match_q_CGCABGCG_t_1_M00652), [AP-2 (M00189)](https://www.cgat.org/downloads/myRInhR8rR/dali/motif_analysis/dali_lacz_input.all.500/dreme_tomtom_out/tomtom.html#match_q_CGCABGCG_t_1_M00189) | AP-2 (Hervouet et al, 2012) |
| 19 |  | DREME | 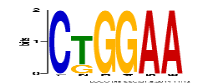 | 7.7e-007 |  | [Pax-6 (M00979)](https://www.cgat.org/downloads/myRInhR8rR/dali/motif_analysis/dali_lacz_input.all.500/dreme_tomtom_out/tomtom.html#match_q_CKGGAA_t_1_M00979), [Pax (M00808)](https://www.cgat.org/downloads/myRInhR8rR/dali/motif_analysis/dali_lacz_input.all.500/dreme_tomtom_out/tomtom.html#match_q_CKGGAA_t_1_M00808), [HSF (M00641)](https://www.cgat.org/downloads/myRInhR8rR/dali/motif_analysis/dali_lacz_input.all.500/dreme_tomtom_out/tomtom.html#match_q_CKGGAA_t_1_M00641) | Pax-6 (Hervouet et al, 2012) |
| 20 |  | DREME | 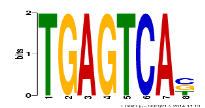 | 2.5e-006 |  | [AP-1 (M00174, M00173, M00926)](https://www.cgat.org/downloads/myRInhR8rR/dali/motif_analysis/dali_lacz_input.all.500/dreme_tomtom_out/tomtom.html#match_q_TGAGTCAB_t_1_M00174) | n/a |
| 21 |  | DREME | 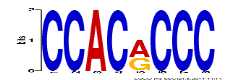 | 3.0e-006 |  | [CACD (M01113)](https://www.cgat.org/downloads/myRInhR8rR/dali/motif_analysis/dali_lacz_input.all.500/dreme_tomtom_out/tomtom.html#match_q_CCACRCCC_t_1_M01113), [EKLF (M01874)](https://www.cgat.org/downloads/myRInhR8rR/dali/motif_analysis/dali_lacz_input.all.500/dreme_tomtom_out/tomtom.html#match_q_CCACRCCC_t_1_M01874), [GKLF (M01588)](https://www.cgat.org/downloads/myRInhR8rR/dali/motif_analysis/dali_lacz_input.all.500/dreme_tomtom_out/tomtom.html#match_q_CCACRCCC_t_1_M01588) | n/a |
| 22 |  | DREME | 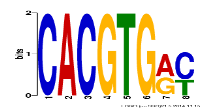 | 2.9e-006 |  | [USF (M00121)](https://www.cgat.org/downloads/myRInhR8rR/dali/motif_analysis/dali_lacz_input.all.500/dreme_tomtom_out/tomtom.html#match_q_CACGTGRY_t_1_M00121), [TFEB (M01768)](https://www.cgat.org/downloads/myRInhR8rR/dali/motif_analysis/dali_lacz_input.all.500/dreme_tomtom_out/tomtom.html#match_q_CACGTGRY_t_1_M01768), [CBF1 (M01793)](https://www.cgat.org/downloads/myRInhR8rR/dali/motif_analysis/dali_lacz_input.all.500/dreme_tomtom_out/tomtom.html#match_q_CACGTGRY_t_1_M01793) | n/a |
| 23 |  | DREME | 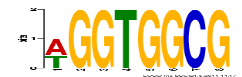 | 2.9e-005 |  | [RPN4 (M01925)](https://www.cgat.org/downloads/myRInhR8rR/dali/motif_analysis/dali_lacz_input.all.500/dreme_tomtom_out/tomtom.html#match_q_CGCCACCW_t_1_M01925), [MyoD (M00929)](https://www.cgat.org/downloads/myRInhR8rR/dali/motif_analysis/dali_lacz_input.all.500/dreme_tomtom_out/tomtom.html#match_q_CGCCACCW_t_1_M00929), [ERF2 (M01057)](https://www.cgat.org/downloads/myRInhR8rR/dali/motif_analysis/dali_lacz_input.all.500/dreme_tomtom_out/tomtom.html#match_q_CGCCACCW_t_1_M01057) | n/a |
| 24 |  | DREME | 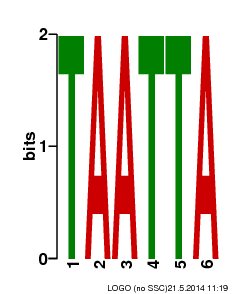 | 3.8e-005 |  | L[hx3a (M00510)](https://www.cgat.org/downloads/myRInhR8rR/dali/motif_analysis/dali_lacz_input.all.500/dreme_tomtom_out/tomtom.html#match_q_TAATTA_t_1_M00510), [IPF1 (M01233)](https://www.cgat.org/downloads/myRInhR8rR/dali/motif_analysis/dali_lacz_input.all.500/dreme_tomtom_out/tomtom.html#match_q_TAATTA_t_1_M01233), [IPF1 (M01235)](https://www.cgat.org/downloads/myRInhR8rR/dali/motif_analysis/dali_lacz_input.all.500/dreme_tomtom_out/tomtom.html#match_q_TAATTA_t_1_M01235) | n/a |
| 25 |  | DREME | 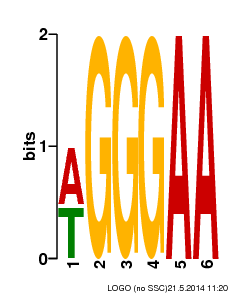 | 6.9e-004 |  | [RBP-Jkappa (M01111)](https://www.cgat.org/downloads/myRInhR8rR/dali/motif_analysis/dali_lacz_input.all.500/dreme_tomtom_out/tomtom.html#match_q_TTCCCW_t_1_M01111), [Ik-3 (M00088)](https://www.cgat.org/downloads/myRInhR8rR/dali/motif_analysis/dali_lacz_input.all.500/dreme_tomtom_out/tomtom.html#match_q_TTCCCW_t_1_M00088) | n/a |
| 26 |  | DREME | 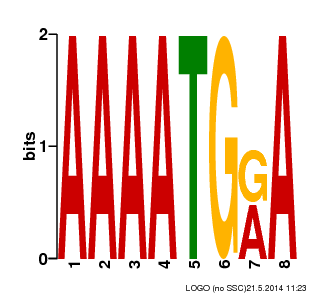 | 1.8e-002 |  | [MADS-A (M00408)](https://www.cgat.org/downloads/myRInhR8rR/dali/motif_analysis/dali_lacz_input.all.500/dreme_tomtom_out/tomtom.html#match_q_AAAATGRA_t_1_M00408), [MADS-B (M00404)](https://www.cgat.org/downloads/myRInhR8rR/dali/motif_analysis/dali_lacz_input.all.500/dreme_tomtom_out/tomtom.html#match_q_AAAATGRA_t_1_M00404), [YY1 (M01894)](https://www.cgat.org/downloads/myRInhR8rR/dali/motif_analysis/dali_lacz_input.all.500/dreme_tomtom_out/tomtom.html#match_q_AAAATGRA_t_1_M01894) | YY1 (Hervouet et al, 2012) |
| 27 |  | DREME | 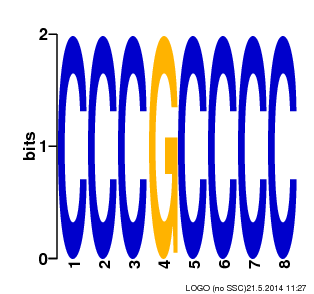 | 2.1e-002 |  | [Sp1 (M00931, M00933, M00196)](https://www.cgat.org/downloads/myRInhR8rR/dali/motif_analysis/dali_lacz_input.all.500/dreme_tomtom_out/tomtom.html#match_q_CCCGCCCC_t_1_M00931) | Sp1 (Hervouet et al, 2012) |
| 28 |  | DREME | 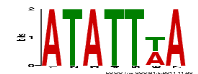 | 2.1e-002 |  | [SIG1 (M01631)](https://www.cgat.org/downloads/myRInhR8rR/dali/motif_analysis/dali_lacz_input.all.500/dreme_tomtom_out/tomtom.html#match_q_ATATTWA_t_1_M01631), [Croc (M00266)](https://www.cgat.org/downloads/myRInhR8rR/dali/motif_analysis/dali_lacz_input.all.500/dreme_tomtom_out/tomtom.html#match_q_ATATTWA_t_1_M00266) | n/a |
